# Medical physics challenges in clinical MR-guided radiotherapy

**DOI:** 10.1186/s13014-020-01524-4

**Published:** 2020-05-05

**Authors:** Christopher Kurz, Giulia Buizza, Guillaume Landry, Florian Kamp, Moritz Rabe, Chiara Paganelli, Guido Baroni, Michael Reiner, Paul J. Keall, Cornelis A. T. van den Berg, Marco Riboldi

**Affiliations:** 1 Department of Radiation Oncology, University Hospital, LMU Munich, Marchioninistraße 15, 81377 Munich, Germany; 2 Department of Medical Physics, Ludwig-Maximilians-Universität München, Am Coulombwall 1, 85748 Garching, Germany; 3 Department of Electronics, Information and Bioengineering, Politecnico di Milano, P.za Leonardo da Vinci 32, 20133 Milano, Italy; 4 German Cancer Consortium (DKTK), 81377 Munich, Germany; 5 Bioengineering Unit, National Center of Oncological Hadrontherapy (CNAO), Strada Privata Campeggi 53, 27100 Pavia, Italy; 6 ACRF Image X Institute, University of Sydney, Sydney, NSW 2006 Australia; 7 Department of Radiotherapy, University Medical Centre Utrecht, PO box 85500, 3508 GA Utrecht, The Netherlands

**Keywords:** Magnetic Resonance Imaging (MRI), image-guided radiotherapy (IGRT), MR-guided radiotherapy (MRgRT), quality assurance (QA), adaptive radiotherapy, quantitative MR imaging (qMRI)

## Abstract

The integration of magnetic resonance imaging (MRI) for guidance in external beam radiotherapy has faced significant research and development efforts in recent years. The current availability of linear accelerators with an embedded MRI unit, providing volumetric imaging at excellent soft tissue contrast, is expected to provide novel possibilities in the implementation of image-guided adaptive radiotherapy (IGART) protocols. This study reviews open medical physics issues in MR-guided radiotherapy (MRgRT) implementation, with a focus on current approaches and on the potential for innovation in IGART.

Daily imaging in MRgRT provides the ability to visualize the static anatomy, to capture internal tumor motion and to extract quantitative image features for treatment verification and monitoring. Those capabilities enable the use of treatment adaptation, with potential benefits in terms of personalized medicine. The use of online MRI requires dedicated efforts to perform accurate dose measurements and calculations, due to the presence of magnetic fields. Likewise, MRgRT requires dedicated quality assurance (QA) protocols for safe clinical implementation.

Reaction to anatomical changes in MRgRT, as visualized on daily images, demands for treatment adaptation concepts, with stringent requirements in terms of fast and accurate validation before the treatment fraction can be delivered. This entails specific challenges in terms of treatment workflow optimization, QA, and verification of the expected delivered dose while the patient is in treatment position. Those challenges require specialized medical physics developments towards the aim of fully exploiting MRI capabilities. Conversely, the use of MRgRT allows for higher confidence in tumor targeting and organs-at-risk (OAR) sparing.

The systematic use of MRgRT brings the possibility of leveraging IGART methods for the optimization of tumor targeting and quantitative treatment verification. Although several challenges exist, the intrinsic benefits of MRgRT will provide a deeper understanding of dose delivery effects on an individual basis, with the potential for further treatment personalization.

## Background

The evolution of delivery techniques in external beam radiotherapy has paralleled the need for online image guidance, aiming at enhanced conformal treatments [1]. The use of technologies such as IMRT (intensity modulated radiotherapy) [2] and VMAT (volumetric modulated arc therapy) [3] has increased the necessity of volumetric imaging as a way to measure and handle uncertainties. In recent years, cone beam CT (CBCT) has progressively become the standard approach to implement IGRT (image-guided radiotherapy) [4, 5]. Nonetheless, CBCT exhibits intrinsic limitations, due to suboptimal image quality, poor soft tissue contrast and the additional imaging dose. Furthermore, the use of CBCT to manage specific uncertainties, such as tumor motion, is not established and may be questioned when considering the inherent shortcomings [6, 7].

For the above-mentioned reasons, magnetic resonance imaging (MRI) has been extensively explored as an alternative to implement IGRT, with significant research and commercial efforts to integrate MRI in treatment delivery devices [8]. MRI has been used for a long time in radiotherapy, with the first application in intracranial radiosurgery published in the mid-1980s, showing the ability to visualize post irradiation changes [9]. Since those pioneering days, MRI has steadily been utilized for more and more cancer sites. The use of MRI for treatment planning simulation in radiation therapy is reported since the early days [10], with first attempts to plan directly on MR images in the 1990s [11]. The increased need for radiotherapy-specific products drove the market for dedicated MRI simulators with flat couch tops, dedicated coils and MRI-compatible immobilization systems.

The increased presence of MRI in radiotherapy, in parallel with the rapid adoption of X-ray and other image guidance methods, spurred the integration of MRI with radiation therapy systems, as a way to implement MRI-based IGRT. An overview of MR-guided radiotherapy (MRgRT) approaches utilizing a linac is shown in Table 1: each of them features different characteristics in terms of magnetic field strength, beam type and energy, and the orientation of the radiation beam and the magnetic field (perpendicular or inline). Among these, the only two commercial systems currently available are the ViewRay MRIdian and the Elekta Unity.

**Table 1:** An overview of linac-based MRgRT approaches. The third column includes information on the field strength and orientation of the magnetic field relative to the radiation beam (perpendicular/inline)

Company/institution	Commercial?	MRI and beam specification	Reference
ViewRay	Yes	0.35 T split bore magnet, 6 MV beam (originally ^60^Co), perpendicular	[12]
Elekta	Yes	1.5 T closed bore magnet, 7 MV beam, perpendicular	[13]
University of Alberta	Under development	0.5 T biplanar magnet, 4 and 6 MV beams, inline/perpendicular	[14]
Australian MR-linac program	No	1.0 T split bore magnet, 4 and 6 MV beams, inline/perpendicular	[15]
Siemens	No	0.5 T closed bore magnet, 6 MV linac inside bore, perpendicular	Patent no. US 8,958,864 B2
Princess Margaret Hospital	No	Separated 1.5 T closed bore magnet on rails and a conventional multi-energy linear accelerator (offline MRgRT)	[16]

MRgRT offers a new paradigm to address delivery uncertainties [12, 17]. While in conventional external beam radiotherapy the patient is positioned to suit a static plan, MRgRT enables adaptation of the plan to optimize dose coverage for the actual patient’s anatomy of the day. Key in this new paradigm is the superb soft tissue contrast of MRI which allows direct visualization of the tumor and the organs-at-risk (OAR) [18]. As an additional benefit, there is no radiation dose burden with MR imaging, which allows for frequent verification. Current advances provide the technological framework to implement IGRT protocols at optimal soft tissue contrast and absence of imaging dose, relying on dedicated procedures for immobilization and imaging. Such imaging capabilities offer the potential for treatment adaptation and quantitative measurement for treatment monitoring and tailoring. Despite such advantages, potential weaknesses exist [19], which require specific medical physics developments to make the most of technological advances in MRI guidance. The intrinsic lack of electron density information, along with the presence of a static magnetic field during treatment delivery, require sophisticated dose calculation methods and dedicated quality assurance (QA) procedures. In addition, MR imaging is prone to spatial distortions and sequence-dependent effects, especially in presence of motion, thus requiring extensive measurements and modeling for accurate MRgRT implementation.

This review will cover the key aspects underlying the necessary physics developments, as a way to summarize the potential and pitfalls of MRgRT. More specifically, this review will address the topics of MR imaging, dose measurement and calculation in the presence of a magnetic field, MR-guided treatment adaptation, as well as QA aspects.

## MR imaging

MR-linacs and in-room MRI scanners allow dose-free extended imaging directly before or during treatment (online) with the patient in treatment position. An MRgRT fraction starts with a so called pre-beam phase, where a first in-room MR image with a relatively large field-of-view (FOV) is acquired to visualize all relevant structures, including OAR and target contours.

### Imaging of the static anatomy

**Fig. 1:**
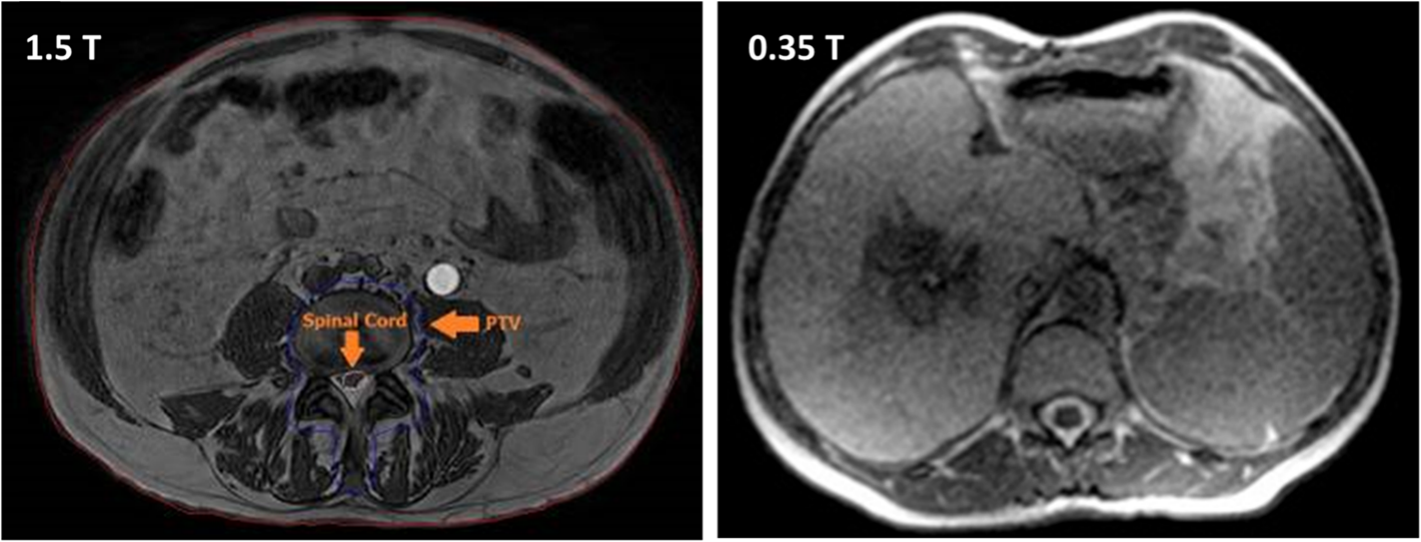
Sample pre-beam images acquired on a 1.5 T MR-linac (3D SPGR sequence, left panel) and on a 0.35 T device (bSSFP sequence, right panel). Adapted and reprinted with permission from [20, 23]

Pre-beam imaging is usually performed using 3D imaging protocols, relying on either stacked 2D slices or native 3D MR acquisition sequences. The former approach has been reported for low-field MR-linacs using a bSSFP (balanced Steady State Free Precession) sequence [20], whereas the latter has been applied for 1.5 T MR-linacs relying on 3D spoiled gradient echo sequences, such as [17, 21], or 3D T2-weighted Turbo Spin Echo (TSE) scans. Native 3D MR acquisition has certain distinct advantages, as it can potentially minimize slice distortions that occur in 2D imaging due to B_0_ inhomogeneity. Furthermore, the 3D isotropic resolution facilitates the verification of the organ contours in all three planes, which might also increase the accuracy of the patient alignment process. Potential optimization in 3D imaging is feasible by the use of 3D T2-weighted TSE imaging, which is characterized by a relatively long echo train with refocusing control, where the flip angle is constantly modulated to find an optimal balance between contrast and sufficient signal during the acquisition of the central k-space lines [22]. Examples of images acquired with low-field and high-field MR-linacs are depicted in Fig. 1.

### Imaging for motion monitoring

In the case of sites affected by respiratory motion, pre-beam imaging in today’s clinical routine is typically performed in breath-hold, utilizing standard 3D sequences. Pre-treatment 4D-MRI might allow for an improved assessment of inter-fractional motion changes [24, 25], but is currently not offered on clinical systems [19], despite the high interest in the development of respiratory-correlated 4D-MRI (rc-4D-MRI) for MRgRT [26]. In rc-4D-MRI, images are created by retrospective sorting or prospective acquisition in image- or k-space domain acquired over several breathing cycles to reconstruct, typically, one average breathing cycle. Fast imaging sequences such as bSSFP, T2-weighted TSE and spoiled gradient echo are most often used for this purpose [26]. The in-plane resolution of the reconstructed 4D-MRI is typically between 1-2 mm with slice thicknesses of around 5 mm for 2D read-out sequences [26] and voxel sizes down to 1.2 × 1.2 × 1.6 mm^3^ [27] for 3D read-out sequences acquired with a diagnostic 1.5 T scanner. The spatial resolution and number of reconstructed breathing phases determines the acquisition time which is in the order of several minutes [26]. Adaptive treatment planning based on rc-4D-MRI could reduce uncertainties compared to today’s clinical standard workflow based on four-dimensional CT [7, 28]. The high-quality images provided by rc-4D-MRI could reduce delineation uncertainties and allow for improved internal target volume [28], mid-position [29] and gating window [26] definitions. As described in the next section, rc-4D-MRI can provide training data for global motion models [30] that could be applied during or after irradiation. Challenges associated with rc-4D-MRI include geometric distortions, long acquisition times [31] and 4D image validation without ground truth data [24, 26]. Furthermore, most rc-4D-MRI methods cannot accurately assess cycle-to-cycle variations and baseline shifts, as typically only one breathing cycle is reconstructed [25].

During irradiation, the beam-on imaging capabilities of today’s clinical MR-linac systems allow real-time intra-fractional monitoring of tumor and OAR motion [12, 32, 33]. Fast 2D cine MRI sequences like bSSFP [34] or spoiled gradient echo [35] achieve acquisition times down to 150 ms [36]. The imaging data is nowadays routinely used for gated treatments of tumors in thoracic and abdominal organs, and first clinical studies have been published [37–40]. These studies used the vendor’s 2D cine bSSFP MRI sequence, acquiring a single sagittal slice with an in-plane resolution of 3.5 × 3.5 mm^2^ and slice thicknesses between 5 and 10 mm at a frame rate of 4 Hz [12, 32, 41].

Research on cine MRI for MRgRT has been focused on the optimization of several sequence parameters. The impact of the spatial resolution on the target tracking accuracy has been investigated by several groups [35, 42, 43]. Robustness of target tracking algorithms and registrations against different geometrical sequence parameters led to the conclusion that only moderate accuracy gains are to be expected from increased imaging resolution with respect to today’s clinical sequences [42, 43]. Better image resolution could still be desirable to improve visibility for smaller lesions, although neighbor structures can be exploited to track reliably. At the same time, different strategies to accelerate image acquisition and reconstruction are investigated in the literature. These include partial Fourier acquisition, different k-space read-out strategies and the use of deep learning to shorten reconstruction times [24, 26]. Parallel imaging capabilities of today’s clinical MR-linacs are also still constrained by the available hardware [25, 41, 44]. The development of faster sequences could enable motion monitoring of the heart, which could be either used to achieve heart dose reduction [45] or to guide cardiac ablation interventions with MR-linacs in the future [46, 47].

The development of new sequences with different imaging contrasts might facilitate image registration and motion tracking and needs to be further investigated [43]. In clinical practice as well as in research studies, the sagittal slice orientation has been most often used for cine MRI [24, 32], but as MR imaging allows for arbitrary slice orientations, the impact of different orientations on the tumor tracking accuracy has been evaluated [25, 43]. No consensus has been found yet [44], but the ideal slice orientation and position might be entity- and patient-specific [43] and depend on the beam angle [48].

As out-of-plane motion can occur when a single 2D slice is used, the acquisition of several parallel slices [12, 49] or the interleaved [50–53] or simultaneous [54, 55] acquisition of orthogonal slices has been investigated. Acquiring imaging data from different orientations could indeed enable the real-time reconstruction of the anatomy in 3D [53]. Based on single or multiple cine MRI slices, the temporally resolved 3D motion of the whole anatomy of the patient can be estimated by global motion models [30, 56, 57]. These models use temporally resolved 1D or 2D surrogate signals as inputs in combination with rc-4D-MRI data that could be acquired in the pre-treatment imaging phase [24]. Up to date, these motion models have not been clinically used for MRgRT and validation with ground truth data remains challenging [30, 58]. Beyond the use of cine MRI for gating, the availability of time-resolved volumetric imaging acquired during treatment will give opportunity to guide multi-leaf collimator (MLC)-tracking [59] and to perform post-beam dose accumulation [26]. This information could be used for real-time image-guided adaptive radiotherapy (IGART) [60] and improved dose-response modelling.

The development of real-time 4D-MRI (rt-4D-MRI) [26, 61–63] with sufficient spatio-temporal resolution would be desirable both for pre-treatment inter-fractional motion characterization as well as for real-time beam-on guidance [24]. Considering the recent advances in imaging and reconstruction acceleration [26] and the observation that coarse spatial resolution could yield acceptable localization errors for real-time MRgRT [42], rt-4D-MRI is expected to play an increasingly important role in MRgRT in the future [26]. As for rc-4D-MRI, geometric distortions and image artifacts of 2D cine MRI and rt-4D-MRI sequences need to be accounted for [64, 65], imaging latencies have to be kept as low as possible [66] and dedicated QA for gating and tracking are needed.

### Quantitative imaging

In radiotherapy-related applications, quantitative MR imaging (qMRI) has been considered to support treatment planning and to implement MRI-only treatment workflows [67, 68]. Specifically, qMRI refers to the objective measurement of a biophysical property of the examined tissue that can be expressed in physical units [69, 70]. Recent efforts in improving qMRI robustness [69, 71] are mainly driven by the clinical need for reliable imaging of biomarkers in oncology [72]. This is not necessarily related to MRgRT, whose clinical implementation is still at an early stage, although qMRI principles could further leverage the importance of MR guidance in the field. Along with opportunities, qMRI also brings new challenges [73] to established clinical practices which may need to be updated; considering for example imaging protocols harmonization, quality control and assurance procedures, staff expertise, data handling and software verification [70, 74–76].

Among qMRI techniques, functional imaging can play a relevant role in MR-guided workflows [77] and its use complemented with that of anatomical acquisition is being explored to provide multi-parametric analyses [78, 79]. Specifically for functional qMRI protocols, dynamic contrast enhanced and diffusion weighted MRI have been widely explored [80–82] due to their sensitivity to vasculature architecture [83] and tissue structure [76, 84], respectively. These sequences could improve any stage of the radiotherapy workflow [75, 85, 86]: from diagnosis and patient stratification [87, 88], through contouring and dose optimization [89, 90], to treatment monitoring and response assessment [83, 91, 92]. Initial clinical experience for diffusion weighted MRI has been reported for the low field MR-linac [93]. In addition, T1 and T2 mapping protocols, obtained through relaxometry [94], can provide quantitative tissue mapping at high spatial resolution. Together with proton density MR, they have been used to improve automatic contouring in radiotherapy [95], to detect early radiation-induced effects [79] or to differentiate recurrent tumors from benign tissue, when coupled to functional qMRI [78].

**Fig. 2:**
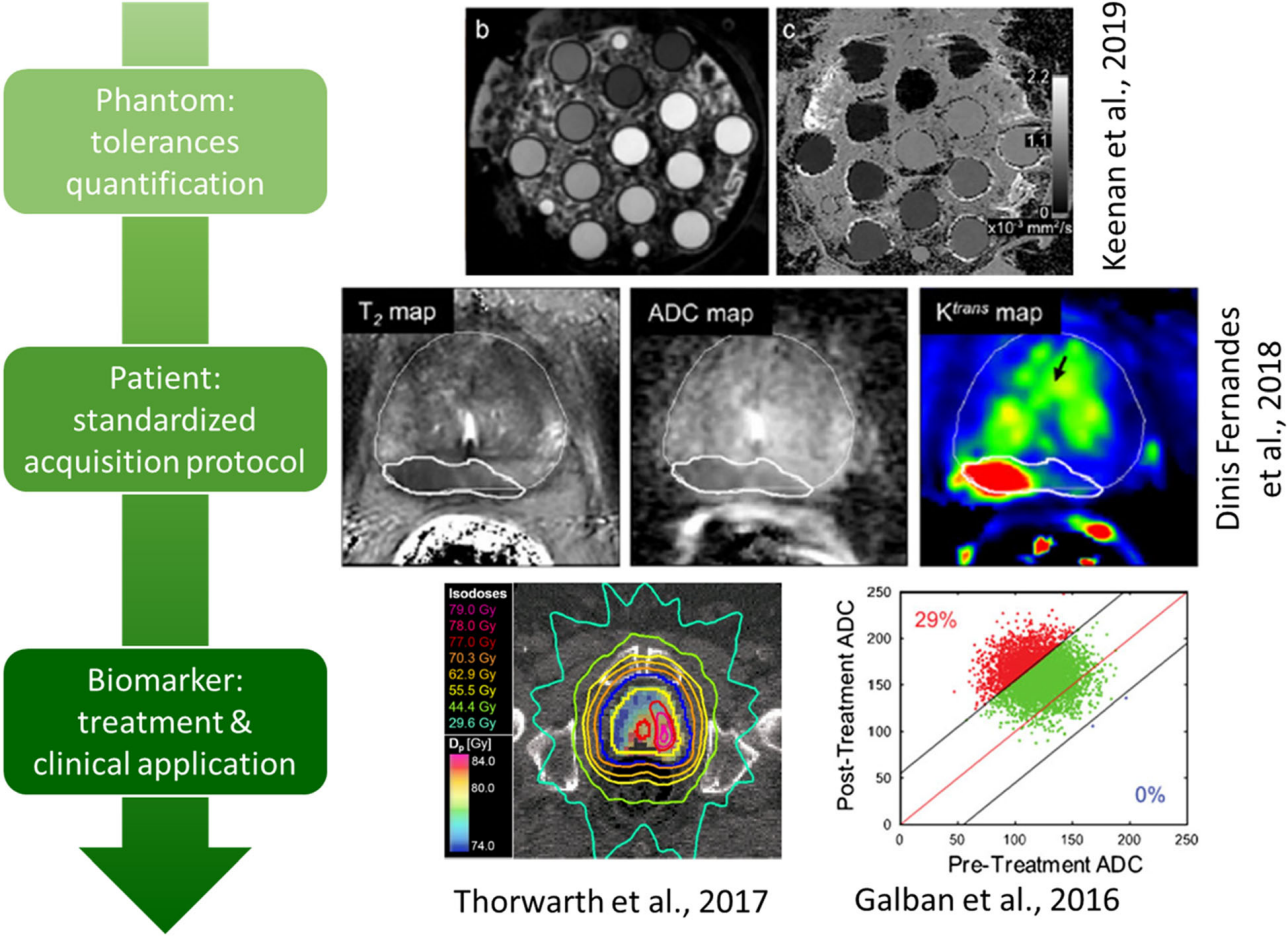
Flowchart for validated use of qMRI biomarkers, with representative images at each step. Adapted and reproduced with permission from [69, 78, 80, 89]

Nevertheless, in order to be reliably employed in the clinical routine, qMRI must undergo both technical and biological/clinical validation [96]. Technical validation in qMRI entails testing for accuracy, repeatability and reproducibility of the underlying physical measurement, both over time and across sites [72, 97]. Technical tolerances related to hardware components should be first identified and then quantified through physical phantoms, by following the available standardized procedures or guidelines and recommendations [71, 98–101]. These latter are being developed and updated by several bodies [69], although a global procedure is yet to be found [67]. At the same time, the analysis of the MR signal itself should be carried out through robust software. Computational methods employed to derive clinically useful quantitative parameters from MRI (i.e., imaging biomarkers [102]) should be validated and their quality assessed by making use of phantoms and reliable open-source tools [71, 99, 103, 104]. On the other hand, the quantification pertaining the biological/clinical validation relies on the evaluation of the relationship between imaging and the underlying biophysical parameter of interest. This relationship must be proven to be quantitative, i.e., accurate, repeatable and reproducible, as well as relevant, specific and consistent [96]. The whole process of validation for qMRI biomarkers is shown in Fig. 2. Overall, quantifying technical and biological tolerances associated to any qMRI protocol is fundamental for computing the minimum detectable variation in the specific qMRI parameter that could, once clinically validated, distinguish underlying pathophysiological changes from measurement uncertainties [71].

The above-mentioned aspects will surely require more resources and optimization efforts, but only by tackling all the variabilities and error sources in the quantitative framework, qMRI can represent a reliable input for MR-guided adaptive radiotherapy.

## Dose measurements and calculations in the presence of magnetic fields

### Dose measurements

Reference dosimetry at MRgRT systems requires modifying the TG51 guidelines [105] from the American Association of Physicists in Medicine (AAPM), or national equivalent (TRS 398 or other), to account for the effects of the magnetic field on i) the depth dose distribution, ii) the ion chamber response, and iii) the impact of the machine design on the definition of reference conditions, such as the source-to-surface distance (SSD). Both i) and ii) are directly related to curved electron paths [106, 107]. In the case of i), these cause an effective reduction of secondary electron range projected along the beam direction, which leads to an upstream shift of both the maximum dose (*d*_*max*_) and the depth dose curve (see Fig. 1 in [108]), thus reducing the dose at the point of measurement by 0.5%/0.3% at 1.5 T/1 T [109, 110]. This means that when asserting the impact on chamber response, care must be taken to account for i), if measuring with and without a magnetic field, and when estimating beam quality specifiers. The change in chamber response ii) is described in the first order by variations in electron trajectories through the sensitive volume; this entails that chamber geometry, orientation and magnetic field strength have a large impact on response [107–113]. However, to fully explain response variations, for example by Monte Carlo modelling, it is necessary to additionally account for the gas volume where charge is collected by the guard electrode [110, 113]. Finite element modelling of electric field lines coupled to Monte Carlo simulation has been shown by [114] to reproduce well chamber response to different field strengths. In practice, chamber response is much less affected when the cylindrical chamber’s axis is parallel to the magnetic field and the latter is perpendicular to the beam. Beam quality specifiers should also be carefully considered in MRgRT systems. *%dd* (10)_*x*_ may be problematic due to non-reference SSDs and the above-mentioned shift in *d*_*max*_; thus most publications recommend the use of ${TPR}_{10}^{20}$, which is not very dependent on SSD [109].

A few modifications to TG51 have been published which allow reference dosimetry at MRgRT systems. O’Brien et al. [109] pioneered the use of a correction to *N*_*D,w*_ in the presence of magnetic fields determined by Monte Carlo simulation of ion chambers. Malkov and Rogers [115] compared the introduction of a correction to *N*_*D,w*_ accounting for both changes in beam quality and presence of the magnetic field, versus an additional correction related only to magnetic field effects. They also use Monte Carlo simulation of ion chambers. Van Asselen et al. [108] proposed to reduce the burden on chamber modelling, which may be desirable given the subtleties of dead volume computation, by proposing a with/without magnetic field measurement-based approach where Monte Carlo simulation is used to determine the change of dose from i) instead. It may however not be feasible for every institution to have the possibility of performing measurements with and without magnetic field, hence the need for consensus values on chamber correction factors for standard ion chamber models in the literature. While the challenges listed above require careful attention, most authors agree that they are not insurmountable and that reference dosimetry is feasible. Particularly with the parallel orientation described above, corrections for chamber response are understood to be well within 1% of unity. Official guidelines for absolute dosimetry are however not currently established, placing a burden on early adopters. Primary standards labs and research groups have also recently worked towards MR-compatible water calorimeters [116–118] and graphite [118, 119] calorimeters. This allows the direct measurement of ion chamber correction factors [120], which should soon lead to additional literature.

For relative dosimetry, care must be taken to account for a shift of the effective point of measurement (EPOM). O’Brien et al. [121] showed, using measurements with and without magnetic field, that at 1.5 T a Farmer chamber exhibits a different EPOM than at 0 T, and recommend using 0.3 × R_cav_. In addition to the depth displacement of the EPOM, lateral displacements are also present, and are detector-dependent. It is expected that at 0.35 T the EPOM displacement is reduced, however, thus far this has not been reported in the literature. Looe et al. [122] used Monte Carlo simulations to show that such shifts are inversely proportional to the detector density.

Commercial cylindrical diode arrays have also been tested at 1.5 T, highlighting a 1.5% maximum dose deviation compared to a standard delivery device [123]. Preliminary studies on the use of thermoluminescent dosimeters have been published [124, 125]. For a 1.5 T field, Matthis et al. reported no difference within a 5% criterion, while Wen et al. reported directional variations of up to 2.3%. Steinmann et al. performed a study of both 0.35 T and 1.5 T systems in anthropomorphic phantoms and have reported deviations of -0.3% and 1.6%, respectively [126].

Due to growing interest in 3D dosimetric detectors, gel and plastic-based solutions have been tested in magnetic fields [127–129]. Polymeric gels have been shown to be comparable to Gafchromic films for checking the isocenter accuracy [128]. Overall, 3D dosimeters for dose response in a magnetic field exhibited a maximum difference of 1.6% in a comparison study [129]. The degradation of plastic 3D dosimeters over time was also quantified, reporting changes in optical density of around 5% per day, which did not impact on IMRT verification relying on AAPM TG 119 [130, 131]. Also, Cherenkov imaging has been tested to acquire high-resolution real-time dosimetric data, though limited to a 2D projection of the 3D dose cube [132].

### Dose calculations

Dose calculation engines designed for treatment planning at MRgRT systems should account for the impact of the magnetic field on the dose from electrons, and be sufficiently fast not to hinder online plan adaptation procedures. The magnetic field will reduce the build-up length, cause a shifted and asymmetric penumbra as well as the electron return effect and may increase skin dose away from the field by deflecting contaminating electrons [106, 133–137]. While Monte Carlo codes such as Geant4, PENELOPE, MCNP and EGSnrc are well benchmarked for conventional radiotherapy, and offer magnetic field capabilities, their emphasis on accuracy renders them unsuitable for treatment planning due to long calculation times. Algorithms making approximations or using variance reduction techniques have thus been developed as an alternative, and been used in conventional treatment planning systems [138–144]. For the 1.5 T Elekta MR-linac, GPUMCD, a GPU-based Monte Carlo engine, has been included in the Monaco TPS and shown to allow fast optimization [143]. GPUMCD uses four tissue classes (air, lung, soft tissue and bone) and a CT number to electron density conversion. For the 0.35 T ViewRay system, the KMC engine has been developed as an improvement to VMC [145–147] to achieve fast calculation speeds. Studies have also been published where research dose calculation engines have been developed and used to compare to the clinical implementations provided by the vendors [148, 149]. Wang et al. developed a GPU-based Monte Carlo simulation platform for the ViewRay MRIdian using ^60^Co [148]. The platform was based on a translation of *penelope* from Fortran to C++, and was called *gpenelope*. They reported calculations times improved by a factor of 152 compared to Penelope, and pass rates for KMC vs gpenelope of 99.1%±0.6% (2%/2 mm). Good agreement with measurements was obtained. Ahmad et al. compared GPUMCD to Geant4 in the presence and absence of a 1.5 T magnetic field [149]. They however did not model the source explicitly and used a point source with a 7 MV spectrum instead. For various combinations of heterogeneities good agreement was observed between Geant4 and GPUMCD.

## MR-guided treatment adaptation

In MRgRT, the baseline treatment plan, optimized based on planning CT and MRI data, can be adapted to the daily anatomical-pathological situation in treatment position as seen on the acquired in-room MRI. The main aim of treatment plan adaptation is to minimize the impact of inter-fractional changes. This enables tighter conformation of the applied dose to the target volume, i.e., practically the use of reduced PTV margins, with optimal sparing of close-by OAR. During irradiation, further measures can be taken to also address intra-fractional changes. The most important steps are outlined in the following.

### Adaptation for inter-fractional changes

**Fig. 3:**
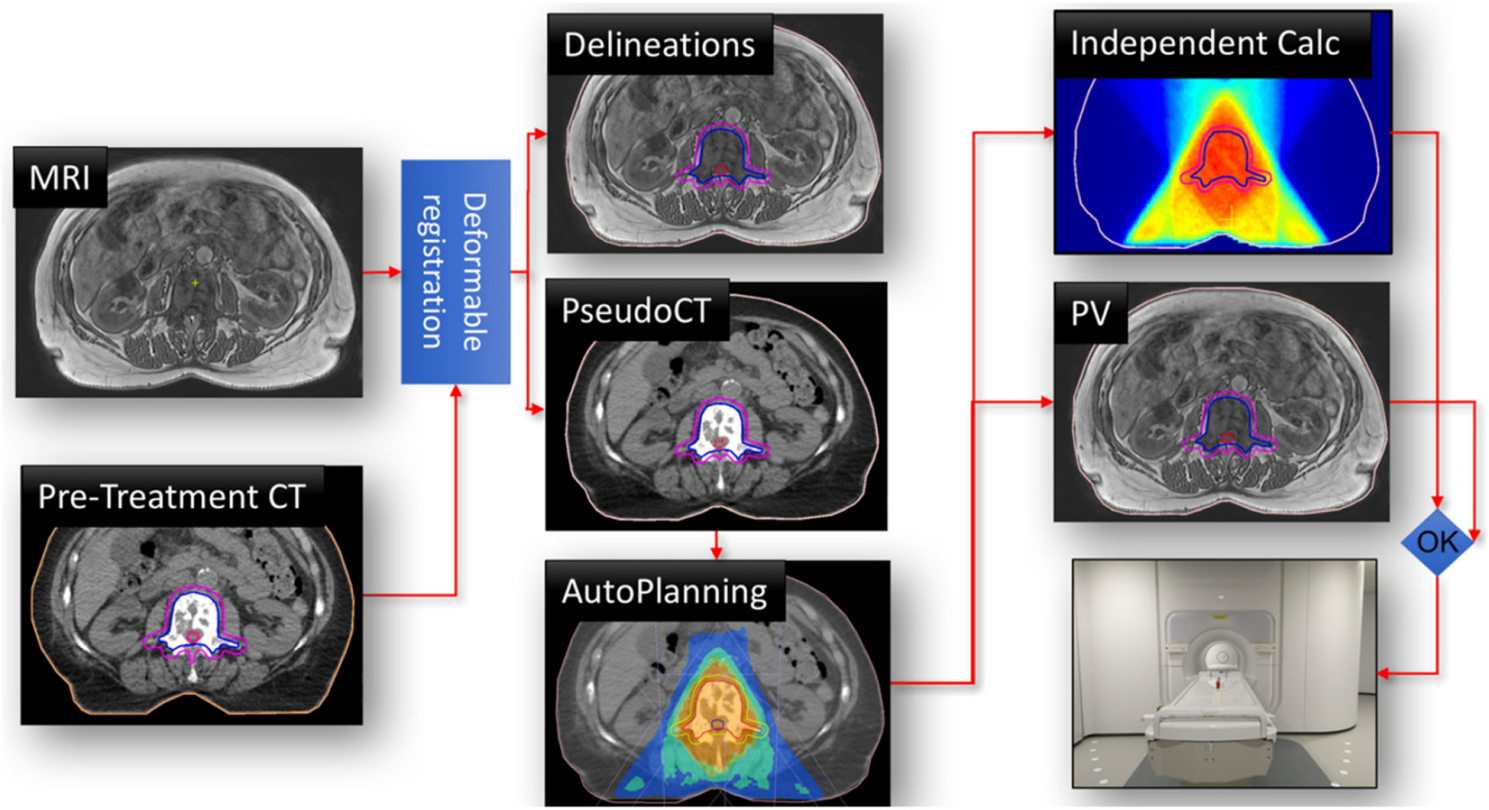
Illustration of an online adaptive MRgRT workflow. The in-room MRI and a pre-treatment CT are used for delineation and pseudo-CT generation. Based on these data, a new treatment plan is optimized (in this case fully automatic). An independent dose calculation is used for online plan QA. In parallel, a final position verification (PV) MRI scan is acquired and eventually the treatment is applied. Reprinted with permission from [17]

On-table re-optimization of the treatment plan generally requires an up-to-date 3D relative electron density image of the patient in treatment position, the corresponding delineation of targets and nearby OAR, fast dose calculation, plan optimization and finally a means to perform fast QA tests on the updated plan. An exemplary workflow is illustrated in Fig. 3.

The typical online adaptive MRgRT workflow starts with patient immobilization and 3D in-room imaging, followed by accurate patient alignment using image fusion. When the patient is positioned correctly, the initial treatment plan is re-calculated on the daily anatomy to infer the dose of the day, which is then used for deciding whether the treatment has to be adapted.

For dose calculation, a 3D electron density map is obtained from pseudo-CT generation methods, typically employing similar methods as developed for PET-MR attenuation correction. While there is a variety of methods for pseudo-CT generation discussed in the literature [150], currently clinically implemented methods rely either directly on deformable image registration (DIR) of a pre-treatment CT [151] or on a combination of DIR and bulk assignment [21]. The reliance on DIR may lead to challenges when the planning CT and in-room MRI exhibit markedly different anatomy. Thus deep learning-based techniques play an increasingly important role for pseudo-CT generation, but are not in clinical use yet [152–155].

Regarding the dose calculation itself, a fast dose engine capable of incorporating magnetic fields is crucial, since plan evaluation and potential re-optimization have to be performed with the patient already in treatment position. Thus, as described above, fast Monte Carlo-based methods are employed for dose calculation [144, 148]

For clinically evaluating the obtained daily dose distribution (and also for potential treatment re-optimization later in the workflow) up-to-date delineation of the target volume and OAR is required. A clear advantage of MRgRT over conventional (CBCT-based) approaches for this task is the superior soft tissue contrast. In clinical online adaptive MRgRT workflows, contour suggestions are obtained from the same DIR used for pseudo-CT generation, followed by manual adaptation by an expert. Since this is still a time-consuming task, typically only a region encompassing the target volume by 2 cm [21] or 3 cm [151] is considered for manual correction due to time constraints. A potential solution overcoming limitations related to DIR, is the use of deep convolutional neural networks that have raised considerable attention for medical image segmentation and recently been applied to in-room MRI data [156–158]. However, clinical certification of such algorithms will be an important hurdle to be cleared in the future.

Considering the daily dose distribution and the updated delineations, a decision whether the treatment plan has to be adapted can be made by the clinical staff, e.g., by inferring deviations in the clinical goals defined at the planning stage. For treatment adaptation, several options exist in the current workflow: for the certified low field MR-linac, adaptation of the table position (3D translation) or full re-optimization are feasible, whereas for the certified high field MR-linac, no table correction is feasible, but the plan has to be adapted instead. Besides full re-optimization of the plan, which might be time-consuming, segment alignment to account for simple target displacement or re-optimization of the segment weights only (since displacing segments in flattening filter free beams may alter the dose rate) are potential alternatives [159]. The re-optimization of the daily treatment plan will take place in an online fashion, why speed, not only in dose calculation, but also in plan optimization is of utmost importance. For this, also auto-planning, promising high and consistent plan quality, might play an increasingly important role in the future [17].

Before applying the adapted plan to the patient, QA of the novel plan has to be performed. For this, independent dose calculations are being performed prior to delivery of the adapted plan and the calculated dose is compared to the prediction of the planning system. More details can be found in the following QA section.

### Adaptation for intra-fractional changes

In current clinical practice, intra-fractional organ motion related to breathing is considered by performing gated beam delivery, potentially in combination with breath-hold techniques to enhance beam-on time [32, 39]. A main advantage in MRgRT is that for gating, the tumor can be directly visualized and tracked using 2D+t cine MRI, as described above. Whenever the delineated tumor moves out of a user-defined gating area, the beam is stopped. While this minimizes the impact of tumor motion on the delivered dose distribution, it can considerably prolong the treatment. Thus, researchers are looking into options for performing real-time treatment adaptation during irradiation to compensate for intra-fractional motion. For this, the tumor could either be continuously trailed during beam delivery [160], or the dose applied up to a certain time point during irradiation could be accumulated (assuming continuous 3D motion monitoring) and used for optimizing the remaining dose to be applied in that treatment fraction [29, 60].

## Quality assurance

In the early clinical adoption phase of MRgRT, QA plays an integral role, but dedicated guidelines and protocols are still missing, often leading to the development of diverse in-house solutions. Thus, early adopters of MRgRT report that sharing experience in QA is desirable to shorten the time needed to reach significant clinical use [161]. In general, a risk assessment based on Failure Mode and Effects Analysis is suggested for setting up required QA procedures. Specific challenges relate to the fact that QA protocols have to be MR-compatible and that proper QA has to carefully address all aspects of MRgRT. These encompass treatment delivery and MRI QA, end-to-end tests, patient-specific QA and QA of the online adaptive workflow.

### Treatment delivery and MRI QA

As early clinical experience in MRgRT is from the ^60^Co system with an integrated 0.35 T MRI [12], the definition of QA procedures relies mostly on that know-how, where online adaptive MRgRT is first documented [162]. Initial procedures for commissioning intensity modulated plan delivery in MRgRT are reported in [131, 163], where the IMRT delivery performance is benchmarked following the recommendations of the AAPM Task Group 119 for IMRT commissioning. In general, characterization and QA of the machine specific beam parameters (percentage depth dose, lateral profiles, flatness, symmetry, output factor, isocenter accuracy and others) closely follow the standard protocol for conventional step-and-shoot IMRT. The same holds true for the obligatory quality checks of the MLC performance (e.g., picket fence test). However, the specific MRgRT setting and presence of the magnetic field have to be considered [164, 165]: typically, conventional water phantoms do not fit in the bore of clinical MRI-linacs and stepping motors are not MR-compatible. Moreover, the orientation of ionization chambers and the corresponding correction factors have to be carefully considered.

On the imaging side, standard quality checks for, among others, B-field homogeneity or signal-to-noise ratio, using conventional MRI QA phantoms (ACR phantom) are crucial. In addition, a critical issue in MRgRT QA is the quantification of spatial distortions induced by MRI [65]. These can be divided into system-dependent and patient-dependent factors [166]. System-dependent distortions are mainly due to static field inhomogeneities and non-linearity effects of the applied magnetic field gradients, where the latter is the dominant effect [166]. Patient-dependent causes include variations in magnetic susceptibility of different tissues [167] and chemical shift, which accounts for intra-tissue deviations due to the surrounding chemical environment [168]. System-related factors, representing the larger uncertainty, are typically handled relying on vendor-specific distortion corrections algorithms, whose performance is dependent on the applied MR imaging sequence and requires verification with geometrical phantoms [169]. Susceptibility induced distortions are more difficult to tackle, as they are strictly dependent on the patient being imaged [166]. It has been shown that major effects are found at the air-tissue interface, with a clear dependency on the magnetic field and gradient strength [166]. Susceptibility effects can be measured using specialized MRI sequences [170] or simulated from an anatomical image, which requires the prior determination of volume susceptibilities of different tissues [171]. Conversely, chemical shift artifacts, mostly visible at the interface of fat regions, can be conveniently reduced by using a wider receiver bandwidth, a smaller FOV, or applying fat saturation techniques in the MR imaging sequence [166].

Finally, yet importantly, dedicated tests ensuring flawless parallel usage of imaging and treatment units have to be performed. Similar to conventional radiotherapy, these tests include verification of accurately aligned imaging and treatment isocenters using dedicated MR-compatible phantoms [172], but also potential system interferences between linac and MRI. To address these issues, Tijssen et al. suggest several dedicated QA tests inferring, among others, image quality at different gantry positions and image quality during beam delivery and MLC movement, which is particularly important when performing imaging during irradiation [65]. Dedicated phantom set-ups and QA procedures for gated beam delivery are also subject of current research [32, 173].

### End-to-end tests

For proper end-to-end QA testing, dedicated phantoms are required. In MRgRT it is crucial that the phantom materials feature not only CT but also MRI visibility [174, 175] for checking all aspects of the treatment workflow, including image fusion and registration as well as potentially plan adaptation and irradiation. Moreover, dosimeters capable of accurate absolute dose measurement in 2D or even 3D in the presence of magnetic fields, e.g., film, dosimetric gel or 3D diode arrays, are required [176–178]. The use of various static and dynamic, geometrical and anthropomorphic phantoms for end-to-end tests is reported, including also dedicated end-to-end tests for stereotactic radiosurgery [179] and for motion management relying on MR guidance [173]. More recently, developments towards specific deformable QA phantoms are reported [175, 180]. Deformable phantoms are of particular interest in MRgRT, since they also enable end-to-end testing of online adaptive workflows, by altering the geometry between planning and irradiation. In addition, efforts on fast, daily end-to-end tests are discussed [181]. Most of these solutions are still dedicated in-house developments and not commercially available.

### Patient-specific QA

Similar to conventional radiotherapy, patient-specific QA protocols dosimetrically verifying the correct irradiation of the optimized treatment plan have been introduced in the MRgRT workflow. Due to the possibility of daily treatment adaptation, not only a single (baseline) treatment plan per patient, but several adapted plans for each patient might have to be verified. Moreover, there is the online aspect, detailed in the following subsection, related to patient-specific QA of the adapted plan prior to irradiation, while the patient is in treatment position. In general, patient-specific baseline plan QA in MRgRT can rely on phantom dose delivery and conventional dose measurement methods (multipoint IC measurement, 2D film dosimetry, quasi 3D diode arrays), similar to conventional radiotherapy [123, 182]. Of course, MR compatibility of dosimeters as well as of the phantoms to be used for dose measurements is crucial. In addition, protocols based solely on machine log files and either 2D fluence verification or 3D Monte Carlo-based dose reconstruction have been reported in the literature [183]. In the case of online plan adaptation there is typically a fast online component and a slower offline component for the plan QA that is performed after completion of the irradiation. Werensteijn-Honingh et al. [21] reported retrospective QA of the adapted plan by means of film dosimetry, while Bertelsen et al. [184] relied on verification by means of a cylindrical diode array in the early clinical adoption phase. Acharya et al. instead performed an offline log-file analysis for adapted plans and only relied on measurements for the baseline plan QA [162]. Also the feasibility of dose reconstruction from EPID measurements during treatment delivery has been shown and might be a future option for patient-specific offline QA of the adapted plans [185].

### Online adaptive workflow QA

In addition to the described offline QA procedures, MRgRT requires specifically designed QA protocols for verifying the online adaptive radiotherapy workflow [186]. The latter implies additional risks with respect to a conventional radiotherapy workflow, as procedures such as image fusion, re-contouring, plan adaptation and plan quality checks need to be performed on the fly. This translates in significant constraints for QA procedures due to limited time availability and the need to check adapted plans with the patient in treatment position [186].

**Fig. 4:**
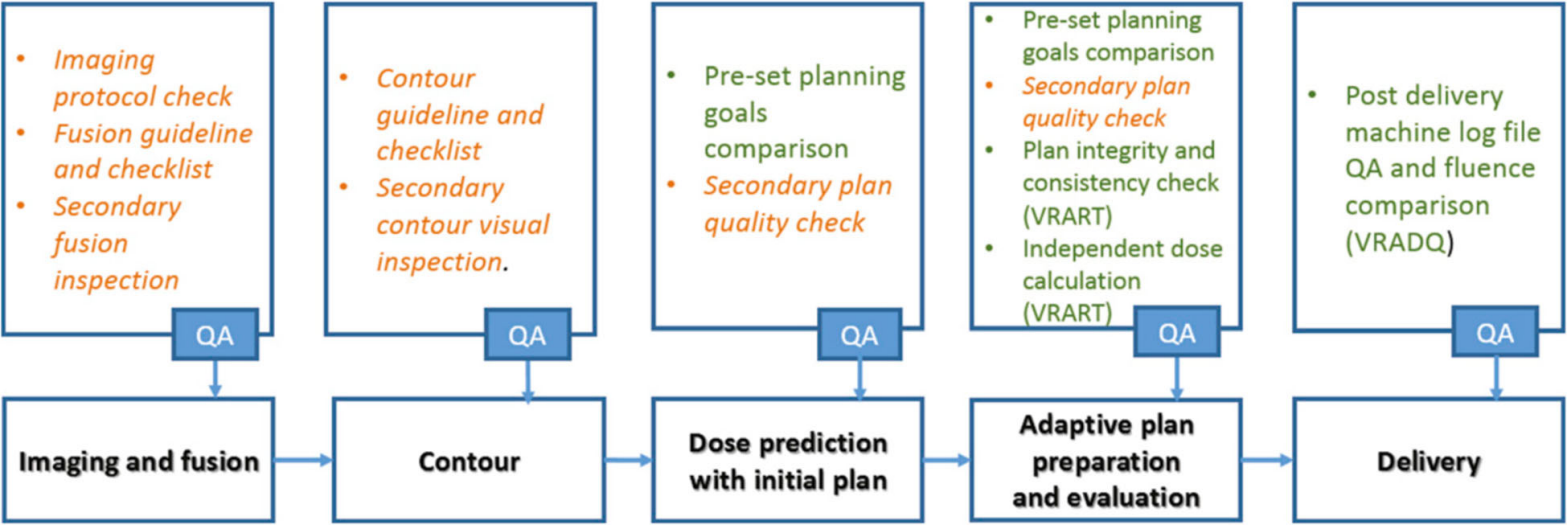
Bottom row – major steps of the online adaptive radiotherapy process for MRgRT. Top row – the associated QA tasks for each step. QA tasks highlighted in orange and italic are the manual checks. QA tasks highlighted in green represent automated checks. The acronyms VRART and VRADQ refer to specific in-house developed software packages. Reprinted with permission from [186]

An example of a QA workflow for online adaptive MRgRT, including both manual and automatic checks, is depicted in Fig. 4. QA starts with checking in-room image acquisition and image fusion. The generated updated contours are verified by dedicated checklists and secondary inspection. Before plan adaptation, the correct settings for treatment plan optimization are ensured and the obtained plan is inspected, e.g., in terms of monitor units, number and shape of segments, but also under consideration of the given clinical goals [41]. Eventually, patient-specific online QA of the adapted plan has to be performed. In today’s clinical practice this is done by means of a secondary independent dose calculation and comparison to the treatment planning system’s dose. For the secondary dose engine, usage of a fast MC-base algorithm [40, 151, 162], but also of a collapsed-cone algorithm (not accounting for the magnetic field) has been reported [17, 21]. As outlined above, the adapted plans are often additionally verified retrospectively by dosimetric measurements or log-file-based dose reconstruction.

## Discussion

MRgRT provides unprecedented capabilities to image the patient before and online during treatment, allowing soft tissue targeting to account for inter- and intra-fractional anatomical variations. In practical clinical use, limitations exist in terms of the optimal trade-off between spatial and temporal resolution for imaging in MRgRT. There are therefore clear constraints in terms of the anatomical detail and/or the time-varying dynamics that the MR image can capture: this typically restricts pre-treatment imaging to static 3D sequences and time-resolved online imaging to single 2D planes in clinical settings. An important medical physics challenge in the near future is the development of fast time-resolved 3D-MRI sequences to improve motion monitoring and compensation in MRgRT. Research is also ongoing in the application of quantitative imaging principles to MRgRT, which would leverage even more the clinical interest in this novel IGART platform. The clinical application of MRgRT is, however, at its initial stages, and the implementation of quantitative imaging protocols will require a more consolidated use to determine the specific areas of interest. Extensive validation is required to fully explore the clinical impact of quantitative information from daily imaging in MRgRT.

Besides imaging, a key challenge in MRgRT application is the realization of dose measurements and dose calculations in the presence of a magnetic field. Conventional measurements techniques require adaption and specific calibration procedures, with a clear impact on QA protocols and medical physics involvement. For neither are dedicated guidelines yet available, driving the early MRgRT adopters to the development of dedicated in-house solutions. MRgRT also emphasizes the need for accurate and independent dose calculation engines, which calls for fast Monte Carlo implementation, as required not only for treatment plan optimization, but also for online plan verification and independent QA. The widespread use of highly efficient hardware platforms and parallel computing programming will definitely contribute to match the requirements for Monte Carlo dose calculations applicable to MRgRT.

Safe clinical adoption of MRgRT also entails unique challenges in terms of QA protocol implementation. Specifically, machine QA and end-to-end tests should, similar to dose measurements, account for the magnetic field, thus increasing the overall complexity. Dedicated tests ensuring the interference-free parallel operation of MRI and linac have to be established. In addition, online QA is required to fully exploit the imaging capabilities for treatment adaptation, with the need to define specific official protocols.

Despite these challenges, the enhanced imaging capabilities offered by MRgRT already enable routine pre-treatment plan adaption as a way to compensate for measured anatomical deviations. This represents a significant step towards more conformal treatments, as, until today, adaptation has mostly been applied via retrospective replanning imaging in reaction to measured deviations in conventional radiotherapy. While pre-treatment adaption is already routinely applied clinically in MRgRT to compensate for inter-fractional changes, intra-fractional changes, e.g., related to breathing motion, are still tackled using gating protocols due to the mentioned limitations in time-resolved imaging. The future development of fast rt-4DMRI protocols would represent an important step towards more efficient motion management by means of real-time 3D tumor and anatomy tracking in combination with real-time online plan adaptation.

Until today, target delineation, dose calculations, plan optimization and QA of the adapted plan in MRgRT contribute to considerably increase the workload with respect to conventional RT. Thus, pre-treatment plan adaptation is naturally better suited to treatments with few fractions, which is why anatomical sites where hypofractionation is indicated, such as prostate [187], pancreas [151], lung [25], liver and adrenal gland [188] as well as oligometastases [21], are preferred. More details on the clinical indications for MRgRT have been recently discussed by Corradini et al. [189]. Both the substantially increased workload, as well as the focus on treatment sites allowing for hypofractionated treatment, limit the routine clinical use of MRgRT. Future medical physics developments streamlining the MRgRT workflow, e.g., in terms of more accurate and robust pseudo-CT generation and improved automatic contour suggestion by means of deep learning, might play an important role to pave the way towards widespread clinical use of MRgRT.

## Conclusions

Recent developments in MRgRT provide effective improvements in soft tissue discrimination and tumor targeting, enabling detection of anatomical changes and treatment adaptation in IGART. The ability to capture and react to inter- and intra-fractional variations is expected to improve our knowledge of dose deposition during treatment. These factors will contribute significantly to our understanding of both tumor as well as normal tissue response. Such advantages come at the price of further complexity in the implementation of effective treatment workflows, with substantial efforts for treatment QA, online evaluation and validation of potential new approaches. Nevertheless, the systematic use of MRI within an IGART workflow brings the potential of better treatment customization and evaluation of the individual treatment response.

## Data Availability

Data sharing is not applicable to this article as no datasets were generated or analyzed during the current study.

## Abbreviations

4D-MRI: Four Dimensional Magnetic Resonance Imaging
bSSFP: balanced Steady State Free Precession
CBCT: Cone beam computed tomography
CT: Computed tomography
DIR: Deformable image registration
*d*_*max*_: Maximum dose
EPOM: Effective point of measurement
FOV: Field-Of-View
IGART: Image-guided adaptive radiation therapy
IGRT: Image-guided radiotherapy
IMRT: Intensity modulated radiation therapy
MRI: Magnetic resonance imaging
MRgRT: MR-guided Radiotherapy
PV: Position verification
OAR: Organs-at-risk
QA: Quality assurance
qMRI: Quantitative MRI
rc-4D-MRI: respiratory-correlated 4D-MRI
rt-4D-MRI: real-time 4D-MRI
SSD: Source-to-surface distance
TSE: Turbo spin echo
VMAT: Volumetric modulated arc therapy

## References

[1] Zou W, Dong L, Kevin Teo BK (2018). Current State of Image Guidance in Radiation Oncology: Implications for PTV Margin Expansion and Adaptive Therapy. Semin Radiat Oncol..

[2] Bortfeld T, Boyer AL, Schlegel W, Kahler DL, Waldron TJ (1994). Realization and verification of three-dimensional conformal radiotherapy with modulated fields. Int J Radiat Oncol Biol Phys..

[3] Otto K (2008). Volumetric modulated arc therapy: IMRT in a single gantry arc. Med Phys..

[4] Landry G, Hua CH (2018). Current state and future applications of radiological image guidance for particle therapy. Med Phys..

[5] Verellen D, De Ridder M, Storme G (2008). A (short) history of image-guided radiotherapy. Radiother Oncol..

[6] Zhang Y, Folkert MR, Li B, Huang X, Meyer JJ, Chiu T, et al. 4D liver tumor localization using cone-beam projections and a biomechanical model. Radiother Oncol. 2018;133:183–92. 10.1016/j.radonc.2018.10.040 PMC6445758 30448003

[7] Steiner E, Shieh CC, Caillet V, Booth J, O'Brien R, Briggs A (2019). Both four-dimensional computed tomography and four-dimensional cone beam computed tomography under-predict lung target motion during radiotherapy. Radiother Oncol..

[8] Liney GP, Whelan B, Oborn B, Barton M, Keall P (2018). MRI-Linear Accelerator Radiotherapy Systems. Clin Oncol (R Coll Radiol)..

[9] Leksell L, Herner T, Leksell D, Persson B, Lindquist C (1985). Visualisation of stereotactic radiolesions by nuclear magnetic resonance. J Neurol Neurosurg Psychiatry..

[10] Thornton AF, Sandler HM, Ten Haken RK, McShan DL, Fraass BA, La Vigne ML (1992). The clinical utility of magnetic resonance imaging in 3-dimensional treatment planning of brain neoplasms. Int J Radiat Oncol Biol Phys..

[11] Schad LR, Bluml S, Hawighorst H, Wenz F, Lorenz WJ (1994). Radiosurgical treatment planning of brain metastases based on a fast, three-dimensional MR imaging technique. Magn Reson Imaging..

[12] Mutic S, Dempsey JF (2014). The ViewRay system: magnetic resonance-guided and controlled radiotherapy. Semin Radiat Oncol..

[13] Lagendijk JJ, Raaymakers BW, van Vulpen M (2014). The magnetic resonance imaging-linac system. Semin Radiat Oncol..

[14] Fallone BG (2014). The rotating biplanar linac-magnetic resonance imaging system. Semin Radiat Oncol..

[15] Keall PJ, Barton M, Crozier S, Australian Mri-Linac Program including contributors from Ingham Institute Illawarra Cancer Care Centre Liverpool Hospital Stanford University Universities of Newcastle Queensland Sydney Western Sydney Wollongong (2014). The Australian magnetic resonance imaging-linac program. Semin Radiat Oncol..

[16] Jaffray DA, Carlone MC, Milosevic MF, Breen SL, Stanescu T, Rink A (2014). A facility for magnetic resonance-guided radiation therapy. Semin Radiat Oncol..

[17] Raaymakers BW, Jurgenliemk-Schulz IM, Bol GH, Glitzner M, Kotte A, van Asselen B, et al. First patients treated with a 1.5 T MRI-Linac: clinical proof of concept of a high-precision, high-field MRI guided radiotherapy treatment. Phys Med Biol. 2017;62(23):L41–50. 10.1088/1361-6560/aa9517 29135471

[18] Lagendijk JJ, Raaymakers BW, Van den Berg CA, Moerland MA, Philippens ME, van Vulpen M (2014). MR guidance in radiotherapy. Phys Med Biol..

[19] van Herk M, McWilliam A, Dubec M, Faivre-Finn C, Choudhury A (2018). Magnetic Resonance Imaging-Guided Radiation Therapy: A Short Strengths, Weaknesses, Opportunities, and Threats Analysis. Int J Radiat Oncol Biol Phys..

[20] Gao Y, Zhou Z, Han F, Cao M, Shaverdian N, Hegde JV (2018). Accelerated 3D bSSFP imaging for treatment planning on an MRI-guided radiotherapy system. Med Phys..

[21] Werensteijn-Honingh AM, Kroon PS, Winkel D, Aalbers EM, van Asselen B, Bol GH (2019). Feasibility of stereotactic radiotherapy using a 1.5T MR-linac: Multi-fraction treatment of pelvic lymph node oligometastases. Radiother Oncol..

[22] Hennig J, Weigel M, Scheffler K (2003). Multiecho sequences with variable refocusing flip angles: optimization of signal behavior using smooth transitions between pseudo steady states (TRAPS). Magn Reson Med..

[23] Winkel D, Bol GH, Kiekebosch IH, Van Asselen B, Kroon PS, Jurgenliemk-Schulz IM (2018). Evaluation of Online Plan Adaptation Strategies for the 1.5T MR-linac Based on “First-In-Man” Treatments. Cureus.

[24] Paganelli C, Whelan B, Peroni M, Summers P, Fast M, van de Lindt T (2018). MRI-guidance for motion management in external beam radiotherapy: current status and future challenges. Phys Med Biol.

[25] Menten MJ, Wetscherek A, Fast MF (2017). MRI-guided lung SBRT: Present and future developments. Phys Med..

[26] Stemkens B, Paulson ES, Tijssen RHN (2018). Nuts and bolts of 4D-MRI for radiotherapy. Phys Med Biol.

[27] Han F, Zhou Z, Cao M, Yang Y, Sheng K, Hu P (2017). Respiratory motion-resolved, self-gated 4D-MRI using rotating cartesian k-space (ROCK). Med Phys..

[28] Thomas DH, Santhanam A, Kishan AU, Cao M, Lamb J, Min Y (2018). Initial clinical observations of intra- and interfractional motion variation in MR-guided lung SBRT. Br J Radiol..

[29] Kontaxis C, Bol GH, Stemkens B, Glitzner M, Prins FM, Kerkmeijer LGW (2017). Towards fast online intrafraction replanning for free-breathing stereotactic body radiation therapy with the MR-linac. Phys Med Biol..

[30] McClelland JR, Hawkes DJ, Schaeffter T, King AP (2013). Respiratory motion models: a review. Med Image Anal..

[31] McGee KP, Hu Y, Tryggestad E, Brinkmann D, Witte B, Welker K (2016). MRI in radiation oncology: Underserved needs. Magn Reson Med..

[32] Green OL, Rankine LJ, Cai B, Curcuru A, Kashani R, Rodriguez V, et al. First clinical implementation of real-time, real anatomy tracking and radiation beam control. Med Phys. 2018;45(8):3728–40. 10.1002/mp.13002 29807390

[33] Jackson S, Glitzner M, Tijssen RHN (2019). Raaymakers BW. MRI B 0 homogeneity and geometric distortion with continuous linac gantry rotation on an Elekta Unity MR-linac. Phys Med Biol.

[34] Bieri O, Scheffler K (2013). Fundamentals of balanced steady state free precession MRI. J Magn Reson Imaging..

[35] Bourque AE, Bedwani S, Carrier JF, Menard C, Borman P, Bos C (2018). Particle Filter-Based Target Tracking Algorithm for Magnetic Resonance-Guided Respiratory Compensation: Robustness and Accuracy Assessment. Int J Radiat Oncol Biol Phys..

[36] Sawant A, Keall P, Pauly KB, Alley M, Vasanawala S, Loo BW (2014). Investigating the feasibility of rapid MRI for image-guided motion management in lung cancer radiotherapy. Biomed Res Int..

[37] Wojcieszynski AP, Rosenberg SA, Brower JV, Hullett CR, Geurts MW, Labby ZE (2016). Gadoxetate for direct tumor therapy and tracking with real-time MRI-guided stereotactic body radiation therapy of the liver. Radiother Oncol..

[38] Henke LE, Contreras JA, Green OL, Cai B, Kim H, Roach MC (2018). Magnetic Resonance Image-Guided Radiotherapy (MRIgRT): A 4.5-Year Clinical Experience. Clin Oncol (R Coll Radiol).

[39] van Sornsen de Koste JR, Palacios MA, Bruynzeel AME, Slotman BJ, Senan S, Lagerwaard FJ (2018). MR-guided Gated Stereotactic Radiation Therapy Delivery for Lung, Adrenal, and Pancreatic Tumors: A Geometric Analysis. Int J Radiat Oncol Biol Phys..

[40] Finazzi T, Palacios MA, Haasbeek CJA, Admiraal MA, Spoelstra FOB, Bruynzeel AME (2019). Stereotactic MR-guided adaptive radiation therapy for peripheral lung tumors. Radiother Oncol..

[41] Kluter S (2019). Technical design and concept of a 0.35 T MR-Linac. Clin Transl Radiat Oncol..

[42] Glitzner M, Denis de Senneville B, Lagendijk J, Raaymakers B, Crijns S (2015). On-line 3D motion estimation using low resolution MRI. Phys Med Biol.

[43] Menten MJ, Fast MF, Wetscherek A, Rank CM, Kachelriess M, Collins DJ (2018). The impact of 2D cine MR imaging parameters on automated tumor and organ localization for MR-guided real-time adaptive radiotherapy. Phys Med Biol..

[44] Bertholet J, Knopf A, Eiben B, McClelland J, Grimwood A, Harris E (2019). Real-time intrafraction motion monitoring in external beam radiotherapy. Phys Med Biol.

[45] McWilliam A, Kennedy J, Hodgson C, Vasquez Osorio E, Faivre-Finn C, van Herk M (2017). Radiation dose to heart base linked with poorer survival in lung cancer patients. Eur J Cancer..

[46] Cuculich PS, Schill MR, Kashani R, Mutic S, Lang A, Cooper D (2017). Noninvasive Cardiac Radiation for Ablation of Ventricular Tachycardia. N Engl J Med..

[47] Ipsen S, Blanck O, Oborn B, Bode F, Liney G, Hunold P (2014). Radiotherapy beyond cancer: target localization in real-time MRI and treatment planning for cardiac radiosurgery. Med Phys..

[48] Wachowicz K, Murray B, Fallone BG (2018). On the direct acquisition of beam's-eye-view images in MRI for integration with external beam radiotherapy. Phys Med Biol..

[49] Ginn JS, Ruan D, Low DA, Lamb JM (2019). Multislice motion modeling for MRI-guided radiotherapy gating. Med Phys..

[50] Bjerre T, Crijns S, af Rosenschold PM, Aznar M, Specht L, Larsen R (2013). Three-dimensional MRI-linac intra-fraction guidance using multiple orthogonal cine-MRI planes. Phys Med Biol..

[51] Tryggestad E, Flammang A, Hales R, Herman J, Lee J, McNutt T (2013). 4D tumor centroid tracking using orthogonal 2D dynamic MRI: implications for radiotherapy planning. Med Phys..

[52] Seregni M, Paganelli C, Lee D, Greer PB, Baroni G, Keall PJ (2016). Motion prediction in MRI-guided radiotherapy based on interleaved orthogonal cine-MRI. Phys Med Biol..

[53] Paganelli C, Lee D, Kipritidis J, Whelan B, Greer PB, Baroni G (2018). Feasibility study on 3D image reconstruction from 2D orthogonal cine-MRI for MRI-guided radiotherapy. J Med Imaging Radiat Oncol..

[54] Mickevicius NJ, Paulson ES (2017). Simultaneous orthogonal plane imaging. Magn Reson Med..

[55] Mickevicius NJ, Paulson ES (2019). Simultaneous acquisition of orthogonal plane cine imaging and isotropic 4D-MRI using super-resolution. Radiother Oncol..

[56] Stemkens B, Tijssen RH, de Senneville BD, Lagendijk JJ, van den Berg CA (2016). Image-driven, model-based 3D abdominal motion estimation for MR-guided radiotherapy. Phys Med Biol..

[57] Garau N, Via R, Meschini G, Lee D, Keall P, Riboldi M (2019). A ROI-based global motion model established on 4DCT and 2D cine-MRI data for MRI-guidance in radiation therapy. Phys Med Biol..

[58] Paganelli C, Portoso S, Garau N, Meschini G, Via R, Buizza G (2019). Time-resolved volumetric MRI in MRI-guided radiotherapy: an in silico comparative analysis. Phys Med Biol..

[59] Glitzner M, Woodhead PL, PTS B, JJW L, Raaymakers BW. MLC-tracking performance on the Elekta unity MRI-linac. Phys Med Biol. 2019;64(15):15NT02. 10.1088/1361-6560/ab2667 31158831

[60] Kontaxis C, Bol GH, Lagendijk JJ, Raaymakers BW (2015). A new methodology for inter- and intrafraction plan adaptation for the MR-linac. Phys Med Biol..

[61] Dinkel J, Hintze C, Tetzlaff R, Huber PE, Herfarth K, Debus J (2009). 4D-MRI analysis of lung tumor motion in patients with hemidiaphragmatic paralysis. Radiother Oncol..

[62] Biederer J, Hintze C, Fabel M, Dinkel J (2010). Magnetic resonance imaging and computed tomography of respiratory mechanics. J Magn Reson Imaging..

[63] Yang YX, Teo SK, Van Reeth E, Tan CH, Tham IW, Poh CL (2015). A hybrid approach for fusing 4D-MRI temporal information with 3D-CT for the study of lung and lung tumor motion. Med Phys..

[64] Ginn JS, Agazaryan N, Cao M, Baharom U, Low DA, Yang Y (2017). Characterization of spatial distortion in a 0.35 T MRI-guided radiotherapy system. Phys Med Biol..

[65] Tijssen RHN, Philippens MEP, Paulson ES, Glitzner M, Chugh B, Wetscherek A (2019). MRI commissioning of 1.5T MR-linac systems - a multi-institutional study. Radiother Oncol..

[66] Borman PTS, Tijssen RHN, Bos C, Moonen CTW, Raaymakers BW, Glitzner M (2018). Characterization of imaging latency for real-time MRI-guided radiotherapy. Phys Med Biol..

[67] Kerkmeijer LGW, Maspero M, Meijer GJ, van der Voort van Zyp JRN, de Boer HCJ, van den Berg CAT (2018). Magnetic Resonance Imaging only Workflow for Radiotherapy Simulation and Planning in Prostate Cancer. Clin Oncol (R Coll Radiol).

[68] Kooreman ES, van Houdt PJ, Nowee ME, van Pelt VWJ, Tijssen RHN, Paulson ES (2019). Feasibility and accuracy of quantitative imaging on a 1.5 T MR-linear accelerator. Radiother Oncol..

[69] Keenan KE, Biller JR, Delfino JG, Boss MA, Does MD, Evelhoch JL, et al. Recommendations towards standards for quantitative MRI (qMRI) and outstanding needs. J Magn Reson Imaging. 2019;49(7):e26–39. 10.1002/jmri.26598 PMC6663309 30680836

[70] Yankeelov TE, Mankoff DA, Schwartz LH, Lieberman FS, Buatti JM, Mountz JM (2016). Quantitative Imaging in Cancer Clinical Trials. Clin Cancer Res..

[71] Shukla-Dave A, Obuchowski NA, Chenevert TL, Jambawalikar S, Schwartz LH, Malyarenko D, et al. Quantitative imaging biomarkers alliance (QIBA) recommendations for improved precision of DWI and DCE-MRI derived biomarkers in multicenter oncology trials. J Magn Reson Imaging. 2018;49(7):e101–21. 10.1002/jmri.26518 PMC6526078 30451345

[72] Morin O, Vallieres M, Jochems A, Woodruff HC, Valdes G, Braunstein SE (2018). A Deep Look Into the Future of Quantitative Imaging in Oncology: A Statement of Working Principles and Proposal for Change. Int J Radiat Oncol Biol Phys..

[73] Winfield JM, Payne GS, Weller A, deSouza NM (2016). DCE-MRI, DW-MRI, and MRS in Cancer: Challenges and Advantages of Implementing Qualitative and Quantitative Multi-parametric Imaging in the Clinic. Top Magn Reson Imaging..

[74] Keenan KE, Ainslie M, Barker AJ, Boss MA, Cecil KM, Charles C (2018). Quantitative magnetic resonance imaging phantoms: A review and the need for a system phantom. Magn Reson Med..

[75] Press RH, Shu HG, Shim H, Mountz JM, Kurland BF, Wahl RL (2018). The Use of Quantitative Imaging in Radiation Oncology: A Quantitative Imaging Network (QIN) Perspective. Int J Radiat Oncol Biol Phys..

[76] Yankeelov TE, Abramson RG, Quarles CC (2014). Quantitative multimodality imaging in cancer research and therapy. Nat Rev Clin Oncol..

[77] Abramson RG, Arlinghaus LR, Dula AN, Quarles CC, Stokes AM, Weis JA (2016). MR Imaging Biomarkers in Oncology Clinical Trials. Magn Reson Imaging Clin N Am..

[78] Dinis Fernandes C, van Houdt PJ, Heijmink S, Walraven I, Keesman R, Smolic M, et al. Quantitative 3T multiparametric MRI of benign and malignant prostatic tissue in patients with and without local recurrent prostate cancer after external-beam radiation therapy. J Magn Reson Imaging. 2018;50(1):269–78. 10.1002/jmri.26581 PMC6618021 30585368

[79] Foltz WD, Wu A, Chung P, Catton C, Bayley A, Milosevic M (2013). Changes in apparent diffusion coefficient and T2 relaxation during radiotherapy for prostate cancer. J Magn Reson Imaging..

[80] Galban CJ, Hoff BA, Chenevert TL, Ross BD. Diffusion MRI in early cancer therapeutic response assessment. NMR Biomed. 2017;30(3). 10.1002/nbm.3458 PMC4947029 26773848

[81] Jaffray DA, Chung C, Coolens C, Foltz W, Keller H, Menard C (2015). Quantitative Imaging in Radiation Oncology: An Emerging Science and Clinical Service. Semin Radiat Oncol..

[82] Leibfarth S, Winter RM, Lyng H, Zips D, Thorwarth D (2018). Potentials and challenges of diffusion-weighted magnetic resonance imaging in radiotherapy. Clin Transl Radiat Oncol..

[83] Quarles CC, Bell LC, Stokes AM. Imaging vascular and hemodynamic features of the brain using dynamic susceptibility contrast and dynamic contrast enhanced MRI. Neuroimage. 2018;187:32–55. 10.1016/j.neuroimage.2018.04.069 PMC6538021 29729392

[84] Tang L, Zhou XJ (2019). Diffusion MRI of cancer: From low to high b-values. J Magn Reson Imaging..

[85] Chandarana H, Wang H, Tijssen RHN, Das IJ (2018). Emerging role of MRI in radiation therapy. J Magn Reson Imaging..

[86] McWilliam A, Rowland B, van Herk M (2018). The Challenges of Using MRI During Radiotherapy. Clin Oncol (R Coll Radiol)..

[87] Shin HJ, Kim HH, Shin KC, Sung YS, Cha JH, Lee JW (2016). Prediction of low-risk breast cancer using perfusion parameters and apparent diffusion coefficient. Magn Reson Imaging..

[88] Wu C, Pineda F, Hormuth DA, Karczmar GS, Yankeelov TE (2019). Quantitative analysis of vascular properties derived from ultrafast DCE-MRI to discriminate malignant and benign breast tumors. Magn Reson Med..

[89] Thorwarth D, Notohamiprodjo M, Zips D, Muller AC (2017). Personalized precision radiotherapy by integration of multi-parametric functional and biological imaging in prostate cancer: A feasibility study. Z Med Phys..

[90] Wang P, Popovtzer A, Eisbruch A, Cao Y (2012). An approach to identify, from DCE MRI, significant subvolumes of tumors related to outcomes in advanced head-and-neck cancer. Med Phys..

[91] Thoeny HC, Ross BD (2010). Predicting and monitoring cancer treatment response with diffusion-weighted MRI. J Magn Reson Imaging..

[92] Buizza G, Molinelli S, D'Ippolito E, Fontana G, Pella A, Valvo F (2019). MRI-based tumour control probability in skull-base chordomas treated with carbon-ion therapy. Radiother Oncol..

[93] Shaverdian N, Yang Y, Hu P, Hart S, Sheng K, Lamb J (2017). Feasibility evaluation of diffusion-weighted imaging using an integrated MRI-radiotherapy system for response assessment to neoadjuvant therapy in rectal cancer. Br J Radiol..

[94] Palma G, Tedeschi E, Borrelli P, Cocozza S, Russo C, Liu S (2015). A Novel Multiparametric Approach to 3D Quantitative MRI of the Brain. PLoS One..

[95] West J, Warntjes JB, Lundberg P (2012). Novel whole brain segmentation and volume estimation using quantitative MRI. Eur Radiol..

[96] O'Connor JP, Aboagye EO, Adams JE, Aerts HJ, Barrington SF, Beer AJ (2017). Imaging biomarker roadmap for cancer studies. Nat Rev Clin Oncol..

[97] Schwartz DL, Tagge I, Powers K, Ahn S, Bakshi R, Calabresi PA, et al. Multisite reliability and repeatability of an advanced brain MRI protocol. J Magn Reson Imaging. 2019;50(3):878–88. 10.1002/jmri.26652 PMC6636359 30652391

[98] Barnes A, Alonzi R, Blackledge M, Charles-Edwards G, Collins DJ, Cook G (2018). UK quantitative WB-DWI technical workgroup: consensus meeting recommendations on optimisation, quality control, processing and analysis of quantitative whole-body diffusion-weighted imaging for cancer. Br J Radiol..

[99] Chao SL, Metens T, Lemort M (2017). TumourMetrics: a comprehensive clinical solution for the standardization of DCE-MRI analysis in research and routine use. Quant Imaging Med Surg..

[100] Ellingson BM, Bendszus M, Boxerman J, Barboriak D, Erickson BJ, Smits M (2015). Consensus recommendations for a standardized Brain Tumor Imaging Protocol in clinical trials. Neuro Oncol..

[101] Fieremans E, Lee HH (2018). Physical and numerical phantoms for the validation of brain microstructural MRI: A cookbook. Neuroimage..

[102] Kessler LG, Barnhart HX, Buckler AJ, Choudhury KR, Kondratovich MV, Toledano A (2015). The emerging science of quantitative imaging biomarkers terminology and definitions for scientific studies and regulatory submissions. Stat Methods Med Res..

[103] Pfaehler E, Zwanenburg A, de Jong JR, Boellaard R (2019). RaCaT: An open source and easy to use radiomics calculator tool. PLoS One..

[104] Smith DS, Li X, Arlinghaus LR, Yankeelov TE, Welch EB (2015). DCEMRI.jl: a fast, validated, open source toolkit for dynamic contrast enhanced MRI analysis. PeerJ.

[105] Almond PR, Biggs PJ, Coursey BM, Hanson WF, Huq MS, Nath R (1999). AAPM's TG-51 protocol for clinical reference dosimetry of high-energy photon and electron beams. Med Phys..

[106] Raaijmakers AJ, Raaymakers BW, van der Meer S, Lagendijk JJ (2007). Integrating a MRI scanner with a 6 MV radiotherapy accelerator: impact of the surface orientation on the entrance and exit dose due to the transverse magnetic field. Phys Med Biol..

[107] Meijsing I, Raaymakers BW, Raaijmakers AJ, Kok JG, Hogeweg L, Liu B (2009). Dosimetry for the MRI accelerator: the impact of a magnetic field on the response of a Farmer NE2571 ionization chamber. Phys Med Biol..

[108] van Asselen B, Woodings SJ, Hackett SL, van Soest TL, Kok JGM, Raaymakers BW (2018). A formalism for reference dosimetry in photon beams in the presence of a magnetic field. Phys Med Biol..

[109] O'Brien DJ, Roberts DA, Ibbott GS, Sawakuchi GO (2016). Reference dosimetry in magnetic fields: formalism and ionization chamber correction factors. Med Phys..

[110] Spindeldreier CK, Schrenk O, Bakenecker A, Kawrakow I, Burigo L, Karger CP (2017). Radiation dosimetry in magnetic fields with Farmer-type ionization chambers: determination of magnetic field correction factors for different magnetic field strengths and field orientations. Phys Med Biol..

[111] Reynolds M, Fallone BG, Rathee S (2013). Dose response of selected ion chambers in applied homogeneous transverse and longitudinal magnetic fields. Med Phys..

[112] Smit K, van Asselen B, Kok JG, Aalbers AH, Lagendijk JJ, Raaymakers BW (2013). Towards reference dosimetry for the MR-linac: magnetic field correction of the ionization chamber reading. Phys Med Biol..

[113] Malkov VN, Rogers DWO (2017). Sensitive volume effects on Monte Carlo calculated ion chamber response in magnetic fields. Med Phys..

[114] Pojtinger S, Kapsch RP, Dohm OS, Thorwarth D (2019). A finite element method for the determination of the relative response of ionization chambers in MR-linacs: simulation and experimental validation up to 1.5 T. Phys Med Biol.

[115] Malkov VN, Rogers DWO (2018). Monte Carlo study of ionization chamber magnetic field correction factors as a function of angle and beam quality. Med Phys..

[116] de Prez L, de Pooter J, Jansen B, Aalbers T (2016). A water calorimeter for on-site absorbed dose to water calibrations in (60) Co and MV-photon beams including MRI incorporated treatment equipment. Phys Med Biol..

[117] de Prez L, de Pooter J, Jansen B, Woodings S, Wolthaus J, van Asselen B (2019). Commissioning of a water calorimeter as a primary standard for absorbed dose to water in magnetic fields. Phys Med Biol..

[118] Renaud J, Sarfehnia A, Bancheri J, Seuntjens J. Absolute dosimetry of a 1.5 T MR-guided accelerator-based high energy photon beam in water and solid phantoms using Aerrow. Med Phys. 2019;47(3):1291–304. 10.1002/mp.13968 31834640

[119] Bancheri J, Seuntjens J, Sarfehnia A, Renaud J (2019). Density effects of silica aerogel insulation on the performance of a graphite probe calorimeter. Med Phys..

[120] de Prez L, Woodings S, de Pooter J, van Asselen B, Wolthaus J, Jansen B (2019). Direct measurement of ion chamber correction factors, k Q and k B, in a 7 MV MRI-linac. Phys Med Biol..

[121] O'Brien DJ, Dolan J, Pencea S, Schupp N, Sawakuchi GO (2018). Relative dosimetry with an MR-linac: Response of ion chambers, diamond, and diode detectors for off-axis, depth dose, and output factor measurements. Med Phys..

[122] Looe HK, Delfs B, Poppinga D, Harder D, Poppe B (2017). Magnetic field influences on the lateral dose response functions of photon-beam detectors: MC study of wall-less water-filled detectors with various densities. Phys Med Biol..

[123] Houweling AC, de Vries JH, Wolthaus J, Woodings S, Kok JG, van Asselen B (2016). Performance of a cylindrical diode array for use in a 1.5 T MR-linac. Phys Med Biol..

[124] Mathis MWZ, Tailor R, Sawakuchi G, Flint D, Beddar S, Ibbott G. SU-E-T-368: effect of a strong magnetic field on select radiation dosimeters. Med Phys. 2014;41(6):309.

[125] ZWJ W, Jiang W, O'Brien D, Sawakuchi G, Ibbott G (2016). SU-G-BRB-08: Investigation On the Magnetic Field Effect On TLDs, OSLDs, and Gafchromic Films Using An MR-Linac. Med Phys..

[126] Steinmann A, O'Brien D, Stafford R, Sawakuchi G, Wen Z, Court L (2019). Investigation of TLD and EBT3 performance under the presence of 1.5T, 0.35T, and 0T magnetic field strengths in MR/CT visible materials. Med Phys..

[127] Alnaghy SJ, Gargett M, Liney G, Petasecca M, Begg J, Espinoza A (2016). Initial experiments with gel-water: towards MRI-linac dosimetry and imaging. Australas Phys Eng Sci Med..

[128] Dorsch S, Mann P, Lang C, Haering P, Runz A, Karger CP (2018). Feasibility of polymer gel-based measurements of radiation isocenter accuracy in magnetic fields. Phys Med Biol.

[129] Lee HJ, Roed Y, Venkataraman S, Carroll M, Ibbott GS (2017). Investigation of magnetic field effects on the dose-response of 3D dosimeters for magnetic resonance - image guided radiation therapy applications. Radiother Oncol..

[130] Mein S, Rankine L, Adamovics J, Li H, Oldham M (2017). Development of a 3D remote dosimetry protocol compatible with MRgIMRT. Med Phys..

[131] Ezzell GA, Burmeister JW, Dogan N, LoSasso TJ, Mechalakos JG, Mihailidis D (2009). IMRT commissioning: multiple institution planning and dosimetry comparisons, a report from AAPM Task Group 119. Med Phys..

[132] Andreozzi JM, Mooney KE, Bruza P, Curcuru A, Gladstone DJ, Pogue BW (2018). Remote Cherenkov imaging-based quality assurance of a magnetic resonance image-guided radiotherapy system. Med Phys..

[133] Raaymakers BW, Raaijmakers AJ, Kotte AN, Jette D, Lagendijk JJ (2004). Integrating a MRI scanner with a 6 MV radiotherapy accelerator: dose deposition in a transverse magnetic field. Phys Med Biol..

[134] Raaijmakers AJ, Raaymakers BW, Lagendijk JJ (2005). Integrating a MRI scanner with a 6 MV radiotherapy accelerator: dose increase at tissue-air interfaces in a lateral magnetic field due to returning electrons. Phys Med Biol..

[135] Kirkby C, Stanescu T, Fallone BG (2009). Magnetic field effects on the energy deposition spectra of MV photon radiation. Phys Med Biol..

[136] Oborn BM, Metcalfe PE, Butson MJ, Rosenfeld AB (2009). High resolution entry and exit Monte Carlo dose calculations from a linear accelerator 6 MV beam under the influence of transverse magnetic fields. Med Phys..

[137] Oborn BM, Metcalfe PE, Butson MJ, Rosenfeld AB (2010). Monte Carlo characterization of skin doses in 6 MV transverse field MRI-linac systems: effect of field size, surface orientation, magnetic field strength, and exit bolus. Med Phys..

[138] Cygler JE, Daskalov GM, Chan GH, Ding GX (2004). Evaluation of the first commercial Monte Carlo dose calculation engine for electron beam treatment planning. Med Phys..

[139] Heath E, Seuntjens J, Sheikh-Bagheri D (2004). Dosimetric evaluation of the clinical implementation of the first commercial IMRT Monte Carlo treatment planning system at 6 MV. Med Phys..

[140] Ma CM, Li JS, Deng J, Fan J (2008). Implementation of Monte Carlo Dose calculationfor CyberKnife treatment planning. J Phys: Conf Ser..

[141] Craig J, Oliver M, Gladwish A, Mulligan M, Chen J, Wong E (2008). Commissioning a fast Monte Carlo dose calculation algorithm for lung cancer treatment planning. J Appl Clin Med Phys..

[142] Hissoiny S, Ozell B, Bouchard H, Despres P (2011). GPUMCD: A new GPU-oriented Monte Carlo dose calculation platform. Med Phys..

[143] Hissoiny S, Raaijmakers AJ, Ozell B, Despres P, Raaymakers BW (2011). Fast dose calculation in magnetic fields with GPUMCD. Phys Med Biol..

[144] Bol GH, Hissoiny S, Lagendijk JJ, Raaymakers BW (2012). Fast online Monte Carlo-based IMRT planning for the MRI linear accelerator. Phys Med Biol..

[145] Kawrakow I, Fippel M, Friedrich K (1996). 3D electron dose calculation using a Voxel based Monte Carlo algorithm (VMC). Med Phys..

[146] Fippel M (1999). Fast Monte Carlo dose calculation for photon beams based on the VMC electron algorithm. Med Phys..

[147] Gardner J, Siebers J, Kawrakow I (2007). Dose calculation validation of Vmc++ for photon beams. Med Phys..

[148] Wang Y, Mazur TR, Green O, Hu Y, Li H, Rodriguez V (2016). A GPU-accelerated Monte Carlo dose calculation platform and its application toward validating an MRI-guided radiation therapy beam model. Med Phys..

[149] Ahmad SB, Sarfehnia A, Paudel MR, Kim A, Hissoiny S, Sahgal A (2016). Evaluation of a commercial MRI Linac based Monte Carlo dose calculation algorithm with GEANT4. Med Phys..

[150] Edmund JM, Nyholm T (2017). A review of substitute CT generation for MRI-only radiation therapy. Radiat Oncol..

[151] Bohoudi O, Bruynzeel AME, Senan S, Cuijpers JP, Slotman BJ, Lagerwaard FJ (2017). Fast and robust online adaptive planning in stereotactic MR-guided adaptive radiation therapy (SMART) for pancreatic cancer. Radiother Oncol..

[152] Han X (2017). MR-based synthetic CT generation using a deep convolutional neural network method. Med Phys..

[153] Maspero M, Savenije MHF, Dinkla AM, Seevinck PR, Intven MPW, Jurgenliemk-Schulz IM (2018). Dose evaluation of fast synthetic-CT generation using a generative adversarial network for general pelvis MR-only radiotherapy. Phys Med Biol..

[154] Dinkla AM, Florkow MC, Maspero M, Savenije MHF, Zijlstra F, Doornaert PAH (2019). Dosimetric evaluation of synthetic CT for head and neck radiotherapy generated by a patch-based three-dimensional convolutional neural network. Med Phys..

[155] Fu J, Yang Y, Singhrao K, Ruan D, Chu FI, Low DA (2019). Deep learning approaches using 2D and 3D convolutional neural networks for generating male pelvic synthetic computed tomography from magnetic resonance imaging. Med Phys..

[156] Fu Y, Mazur TR, Wu X, Liu S, Chang X, Lu Y (2018). A novel MRI segmentation method using CNN-based correction network for MRI-guided adaptive radiotherapy. Med Phys..

[157] Spieler B, Patel NV, Breto AL, Ford J, Stoyanova R, Zavala-Romero O (2019). Automatic Segmentation of Abdominal Anatomy by Artificial Intelligence (AI) in Adaptive Radiotherapy of Pancreatic Cancer. Int J Radiat Oncol Biol Phys..

[158] Eppenhof KAJ, Maspero M, Savenije MHF, de Boer JCJ, van der Voort van Zyp JRN, Raaymakers BW, et al. Fast contour propagation for MR-guided prostate radiotherapy using convolutional neural networks. Med Phys. 2019;47(3):1238–48. 10.1002/mp.13994 PMC7079098 31876300

[159] Winkel D, BGH W-HIAM, Kiekebosch IH, van Asselen B, Intven MPW, Epping WSC, Raaymakers BW, Jürgenliemk-Schulz IM, Kroon PS (2019). Evaluation of plan adaptation strategies for stereotactic radiotherapy of lymph node oligometastases using online magnetic resonance image guidance. Physics Imaging Radiat Oncol.

[160] Fast M, van de Schoot A, van de Lindt T, Carbaat C, van der Heide U, Sonke JJ (2019). Tumor Trailing for Liver SBRT on the MR-Linac. Int J Radiat Oncol Biol Phys..

[161] Kerkmeijer LG, Fuller CD, Verkooijen HM, Verheij M, Choudhury A, Harrington KJ (2016). The MRI-Linear Accelerator Consortium: Evidence-Based Clinical Introduction of an Innovation in Radiation Oncology Connecting Researchers, Methodology, Data Collection, Quality Assurance, and Technical Development. Front Oncol..

[162] Acharya S, Fischer-Valuck BW, Kashani R, Parikh P, Yang D, Zhao T (2016). Online Magnetic Resonance Image Guided Adaptive Radiation Therapy: First Clinical Applications. Int J Radiat Oncol Biol Phys..

[163] Wooten HO, Rodriguez V, Green O, Kashani R, Santanam L, Tanderup K (2015). Benchmark IMRT evaluation of a Co-60 MRI-guided radiation therapy system. Radiother Oncol..

[164] Cho JD, Park JM, Choi CH, Kim J, Wu H, Park S (2017). Implementation of AAPM’s TG-51 Protocol on Co-60 MRI-Guided Radiation Therapy System. Prog Med Phys..

[165] Smit K, Sjoholm J, Kok JG, Lagendijk JJ, Raaymakers BW (2014). Relative dosimetry in a 1.5 T magnetic field: an MR-linac compatible prototype scanning water phantom. Phys Med Biol..

[166] Weygand J, Fuller CD, Ibbott GS, Mohamed AS, Ding Y, Yang J (2016). Spatial Precision in Magnetic Resonance Imaging-Guided Radiation Therapy: The Role of Geometric Distortion. Int J Radiat Oncol Biol Phys..

[167] Stanescu T, Wachowicz K, Jaffray DA (2012). Characterization of tissue magnetic susceptibility-induced distortions for MRIgRT. Med Phys..

[168] Hood MN, Ho VB, Smirniotopoulos JG, Szumowski J (1999). Chemical shift: the artifact and clinical tool revisited. Radiographics..

[169] Karger CP, Hoss A, Bendl R, Canda V, Schad L (2006). Accuracy of device-specific 2D and 3D image distortion correction algorithms for magnetic resonance imaging of the head provided by a manufacturer. Phys Med Biol..

[170] Emmerich J, Laun FB, Pfaffenberger A, Schilling R, Denoix M, Maier F (2018). Technical Note: On the size of susceptibility-induced MR image distortions in prostate and cervix in the context of MR-guided radiation therapy. Med Phys..

[171] Jonsson JH, Garpebring A, Karlsson MG, Nyholm T (2012). Internal fiducial markers and susceptibility effects in MRI-simulation and measurement of spatial accuracy. Int J Radiat Oncol Biol Phys..

[172] Dorsch S, Mann P, Elter A, Runz A, Spindeldreier CK, Kluter S (2019). Measurement of isocenter alignment accuracy and image distortion of an 0.35 T MR-Linac system. Phys Med Biol.

[173] Schneider S, Dolde K, Engler J, Hoffmann A, Pfaffenberger A (2019). Commissioning of a 4D MRI phantom for use in MR-guided radiotherapy. Med Phys..

[174] Steinmann A, Stafford RJ, Sawakuchi G, Wen Z, Court L, Fuller CD (2018). Developing and characterizing MR/CT-visible materials used in QA phantoms for MRgRT systems. Med Phys..

[175] Niebuhr NI, Johnen W, Echner G, Runz A, Bach M, Stoll M (2019). The ADAM-pelvis phantom-an anthropomorphic, deformable and multimodal phantom for MRgRT. Phys Med Biol.

[176] Ahunbay EE, Chen X, Paulson ES, Chen GP, Li A (2018). An End-to-End Verification of Online Adaptation Process on a High-Field MR-Linac. Int J Radiat Oncol Biol Phys..

[177] Hoffmans D, Bohoudi O, Niebuhr N, Pfaffenberger A, Battum L, Slotman B (2018). OC-0409: A film-based end-to-end test for MR-guided online adaptive radiotherapy. Radiother Oncol..

[178] Pappas E, Kalaitzakis G, Boursianis T, Zoros E, Zourari K, Pappas EP (2019). Dosimetric performance of the Elekta Unity MR-linac system: 2D and 3D dosimetry in anthropomorphic inhomogeneous geometry. Phys Med Biol..

[179] Wen N, Kim J, Doemer A, Glide-Hurst C, Chetty IJ, Liu C (2018). Evaluation of a magnetic resonance guided linear accelerator for stereotactic radiosurgery treatment. Radiother Oncol..

[180] Elter A, Dorsch S, Mann P, Runz A, Johnen W, Spindeldreier CK (2019). End-to-end test of an online adaptive treatment procedure in MR-guided radiotherapy using a phantom with anthropomorphic structures. Phys Med Biol..

[181] Chen X, Ahunbay E, Paulson ES, Chen G, Li XA. A daily end-to-end quality assurance workflow for MR-guided online adaptive radiation therapy on MR-Linac. J Appl Clin Med Phys. 2019;21(1):205–12. 10.1002/acm2.12786 PMC6964761 31799753

[182] Barten DLJ, Hoffmans D, Palacios MA, Heukelom S, van Battum LJ (2018). Suitability of EBT3 GafChromic film for quality assurance in MR-guided radiotherapy at 0.35 T with and without real-time MR imaging. Phys Med Biol.

[183] Li HH, Rodriguez VL, Green OL, Hu Y, Kashani R, Wooten HO (2015). Patient-specific quality assurance for the delivery of (60) Co intensity modulated radiation therapy subject to a 0.35-T lateral magnetic field. Int J Radiat Oncol Biol Phys..

[184] Bertelsen AS, Schytte T, Moller PK, Mahmood F, Riis HL, Gottlieb KL (2019). First clinical experiences with a high field 1.5 T MR linac. Acta Oncol..

[185] Torres-Xirau I, Olaciregui-Ruiz I, van der Heide UA, Mans A (2019). Two-dimensional EPID dosimetry for an MR-linac: Proof of concept. Med Phys..

[186] Cai B, Green OL, Kashani R, Rodriguez VL, Mutic S, Yang D (2018). A practical implementation of physics quality assurance for photon adaptive radiotherapy. Z Med Phys..

[187] Pathmanathan AU, van As NJ, Kerkmeijer LGW, Christodouleas J, Lawton CAF, Vesprini D (2018). Magnetic Resonance Imaging-Guided Adaptive Radiation Therapy: A “Game Changer” for Prostate Treatment?. Int J Radiat Oncol Biol Phys..

[188] Henke L, Kashani R, Robinson C, Curcuru A, DeWees T, Bradley J (2018). Phase I trial of stereotactic MR-guided online adaptive radiation therapy (SMART) for the treatment of oligometastatic or unresectable primary malignancies of the abdomen. Radiother Oncol..

[189] Corradini S, Alongi F, Andratschke N, Belka C, Boldrini L, Cellini F (2019). MR-guidance in clinical reality: current treatment challenges and future perspectives. Radiat Oncol..

